# Clinical-Based vs. Model-Based Adaptive Dosing Strategy: Retrospective Comparison in Real-World mRCC Patients Treated with Sunitinib

**DOI:** 10.3390/ph14060494

**Published:** 2021-05-24

**Authors:** Florent Ferrer, Jonathan Chauvin, Bénédicte DeVictor, Bruno Lacarelle, Jean-Laurent Deville, Joseph Ciccolini

**Affiliations:** 1 SMARTc Unit, Centre de Recherche en Cancérologie de Marseille, Inserm U1068 Aix Marseille Université, 13385 Marseille, France; florent-ferrer@sfr.fr (F.F.); bruno.lacarelle@ap-hm.fr (B.L.); 2 Laboratoire de Pharmacocinétique et Toxicologie, La Timone University Hospital of Marseille, 13385 Marseille, France; benedicte.devictor@ap-hm.fr; 3 LiXoft, 92160 Antony, France; jonathan.chauvin@lixoft.com; 4 Medical Oncology Unit, La Timone University Hospital of Marseille, 13385 Marseille, France; Jean-laurent.DEVILLE@ap-hm.fr

**Keywords:** sunitinib, oral targeted therapy, oncology, pharmacokinetics, PK/PD modeling, therapeutic drug monitoring, model-based adaptive dosing, precision medicine

## Abstract

Different target exposures with sunitinib have been proposed in metastatic renal cell carcinoma (mRCC) patients, such as trough concentrations or AUCs. However, most of the time, rather than therapeutic drug monitoring (TDM), clinical evidence is preferred to tailor dosing, i.e., by reducing the dose when treatment-related toxicities show, or increasing dosing if no signs of efficacy are observed. Here, we compared such empirical dose adjustment of sunitinib in mRCC patients, with the parallel dosing proposals of a PK/PD model with TDM support. In 31 evaluable patients treated with sunitinib, 53.8% had an empirical change in dosing after treatment started (i.e., 46.2% decrease in dosing, 7.6% increase in dosing). Clinical benefit was observed in 54.1% patients, including 8.3% with complete response. Overall, 58.1% of patients experienced treatment discontinuation eventually, either because of toxicities or progressive disease. When choosing 50–100 ng/mL trough concentrations as a target exposure (i.e., sunitinib + active metabolite N-desethyl sunitinib), 45% patients were adequately exposed. When considering 1200–2150 ng/mL.h as a target AUC (i.e., sunitinib + active metabolite N-desethyl sunitinib), only 26% patients were in the desired therapeutic window. TDM with retrospective PK/PD modeling would have suggested decreasing sunitinib dosing in a much larger number of patients as compared with empirical dose adjustment. Indeed, when using target trough concentrations, the model proposed reducing dosing for 61% patients, and up to 84% patients based upon target AUC. Conversely, the model proposed increasing dosing in 9.7% of patients when using target trough concentrations and in 6.5% patients when using target AUC. Overall, TDM with adaptive dosing would have led to tailoring sunitinib dosing in a larger number of patients (i.e., 53.8% vs. 71–91%, depending on the chosen metrics for target exposure) than a clinical-based decision. Interestingly, sunitinib dosing was empirically reduced in 41% patients who displayed early-onset severe toxicities, whereas model-based recommendations would have immediately proposed to reduce dosing in more than 80% of those patients. This observation suggests that early treatment-related toxicities could have been partly avoided using prospective PK/PD modeling with adaptive dosing. Conversely, the possible impact of model-based adapted dosing on efficacy could not be fully evaluated because no clear relationship was found between baseline exposure levels and sunitinib efficacy measured at 3 months.

## 1. Introduction

Sunitinib is an oral, multi-target tyrosine kinase inhibitor targeting vascular endothelial growth factor receptor (VEGFR) and platelet-derived growth factor receptor (PDGFR) [1]. It is approved as first line treatment for metastatic renal clear cell carcinoma (mRCC) [2]. Sunitinib’s safety profile is considered to be a concern, with several adverse events impairing quality of life and leading to frequent treatment discontinuation, or empirical dose reductions [2,3,4,5]. In order to improve the efficacy/toxicity balance of sunitinib, alternate treatment scheduling has been empirically tested beyond the standard scheme (i.e., 50 mg QD 4 weeks on/2 weeks off, also known as 4/2 scheduling), such as 18.5 mg continuous dosing, 37.5 mg 4/2 scheduling or more recently 50 mg QD 2/1 scheduling [6]. Sunitinib PK/PD relationships have been extensively studied and different therapeutic windows have been proposed. For instance, the total residual concentration of sunitinib and its active metabolite N-desethyl sunitinib (SU12662) in plasma should be ≥50 ng/mL to inhibit VEGFR and PDGFR and achieve some antitumor efficacy [1]. Additionally, plasma concentrations >100 ng/mL are usually associated with higher risk for dose-limiting toxicities [3]. Several studies have similarly tried to determine a target AUC, and plasma exposure between 1200 and 2150 ng/mL.h has been proposed to ensure therapeutic efficacy [7]. Therapeutic drug monitoring (TDM) is a strategy more and more frequently used in routine clinical settings to monitor the observance and to ensure that drug levels are in the target range of concentrations, especially with oral targeted therapies [8,9,10]. Due to the many causes impacting on sunitinib pharmacokinetics variability [5], implementing TDM with sunitinib and active metabolite N-desethyl sunitinib is an appealing strategy [11]. Shifting towards more personalized medicine in oncology would require the use of dedicated PK/PD models. Such models would enable calculating individual PK parameters to determine the best dosing associated with the desired target exposure. Here, we have retrospectively tested such a PK/PD model on 33 routine patients (31 fully evaluable) treated with standard sunitinib in our institute, and compared empirical dose reduction with the model’s recommendations. The objective of this work was primarily to determine whether such a model could have helped in avoiding early-onset toxicities or treatment failure in mRCC patients treated with sunitinib.

## 2. Results

### 2.1. Patients and Treatments

**Figure 1:**
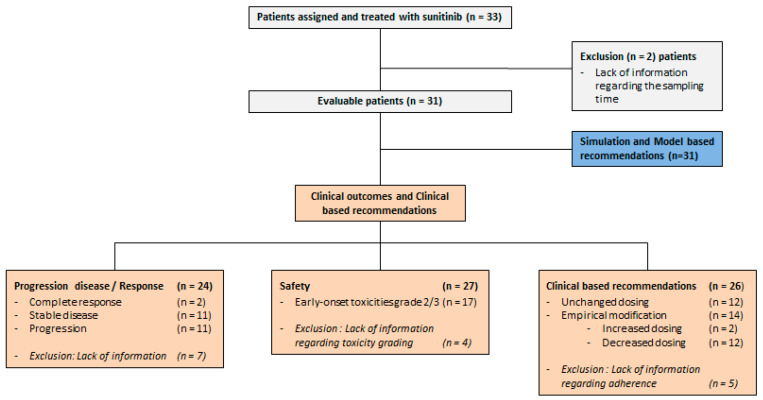
Flowchart of the observational study.

Overall, 33 mRCC adult patients were treated with sunitinib in our institute during this observational study. However, only 31 patients (i.e., 94%) could be fully evaluated (Figure 1) in this study because of a lack of information regarding the sampling time for two patients, preventing PK modeling and adaptive dosing from being properly performed. Patients’ demographic and clinical characteristics, as well as initial dosing and scheduling of sunitinib, are summarized in Table 1. The median follow-up period was 10.6 months (range: 1.9–43.2).

### 2.2. Clinical Outcome

Out of the 31 patients, clinical benefit could be evaluated in only 24 patients. Indeed, seven patients were lost to follow-up before response evaluation and had to be removed from the final evaluation for clinical response.

Clinical benefit was observed in 13 patients (54.1%). Two patients (i.e., 8.3%) achieved complete response, 11 (45.8%) had stable disease, 11 (45.8%) had progressive disease.

Four of the seven patients lost to follow-up also had a lack of clear information concerning safety data (i.e., no precise grading for the non-hematological toxicities in hospital records) and thus could not be properly evaluated for sunitinib-related toxicity.

When considering early-onset toxicities (i.e., side effects showing from the first month after treatment initiation), 17 out of the 27 evaluable patients (i.e., 63%) experienced at least one grade 2 or grade 3 toxicity. The remaining patients had either no toxicities or only grade 1 side effects. The most common grade 3 toxicities were diarrhoea (18.5%) and arterial hypertension (14.8%) (Table 2). No grade 4 toxicity was observed.

### 2.3. Clinical-Based Dose Tailoring and Treatment Discontinuation

Out of the 31 patients treated during the observation period, five were not evaluable due to lack of information regarding the exact changes in sunitinib dosing, and possible concerns about adherence. Consequently, only 26 patients were fully monitored and could be finally analyzed.

During the observation period, 14 out of the remaining 26 patients (i.e., 53.8%) required empirical dose modifications upon clinical signs: 12 (46.2%) had their dosing cut because of the severity of adverse events, and two patients (7.6%) had increased dosing because of signs of a lack of efficacy (e.g., reported pain during the drug-free intervals). Conversely, 12 patients (46.2%) had their initial sunitinib dosing maintained throughout. Overall, 18 of 31 patients (58.1%) discontinued sunitinib eventually, mostly due to adverse events (n = 5, 16.1%), disease progression (n = 8, 25.8%), complete response (n = 2, 6.5%) or a switch to radiotherapy (n = 3, 9.7%).

### 2.4. Model Simulations for Trough Concentrations and AUC

**Figure 2:**
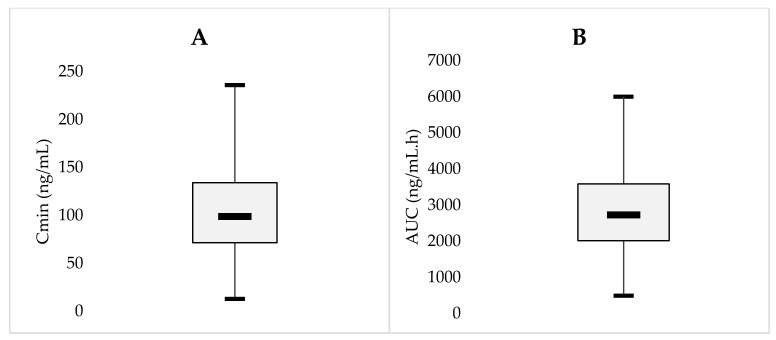
(**A**,**B**) Inter-individual variability among patients in Cmin (**A**) and AUC_T, SS_ (**B**) values.

Overall, mean trough concentrations (either actually measured or simulated) at baseline were 108 ± 49 ng/mL (range: 13–236, coefficient of variation (C.V.): 45%). Mean calculated AUCs were 2927 ± 1223 ng/mL (range: 491–5998, C.V.: 42%). Figure 1A,B show the variability in Cmin and AUC values recorded among patients (Figure 2).

When considering target trough concentrations, 14 out of 31 patients (45.2%) had measured or simulated trough concentrations within the 50–100 ng/mL range at baseline. Seventeen patients (54.8%) were therefore out of the 50–100 ng/mL range, 2 (6.5%) having trough concentrations below the target, whereas, conversely, 15 patients (48.3 =%) were overexposed with respect to target values.

When considering target AUCs, eight out of 31 patients (25.8%) had simulated drug exposure levels (AUC) within the 1200–2150 ng/mL.h range. Out of the 23 remaining patients (74.2%), only one (3.2%) was underexposed whereas 22 patients (71%) were overexposed.

When comparing target trough concentrations and target AUC, a perfect match was observed for 23 (74.2%) patients, i.e., patients with trough concentrations above the target showed AUCs above the target and vice versa. Oppositely, in eight patients (25.8%), discordant results were observed, with the simulated AUC being always slightly higher, i.e., patients with normal trough concentrations showed AUCs above 2150 ng/mL.h, or patients with trough concentrations below the 50 ng/mL target showed AUCs in the 1200 to 2150 ng/mL.h range.

### 2.5. Sunitinib Exposure and Clinical Outcome

**Figure 3:**
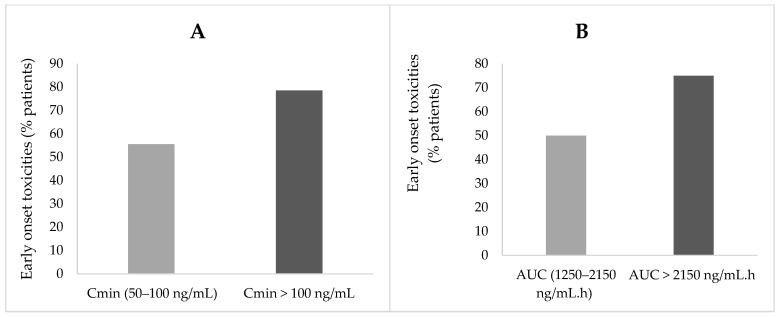
(**A**,**B**) Evaluation of early-onset toxicities (≥ Grade 2) as a function of trough level concentrations (Cmin) (**A**) and exposure (AUC) (**B**).

When considering early-onset toxicities, a trend was observed between exposure levels higher than the respective targets for Cmin or AUC and a higher occurrence of severe toxicities (Figure 3). Grade 2 and grade 3 toxicities were observed in 56% of patients (five out of nine patients) with trough concentrations were in the 50–100 ng/mL range, and in 79% of patients (11 out of 14 patients) with Cmin >100 ng/mL. Similarly, grade 2 and above toxicities were found in 50% of patients (two out of four patients) with AUCs from 1250–2150 ng/mL.h, but in 75% of patients (15 out of 20 patients) with AUCs higher than 2150 ng/mL.h. However, those differences were not great enough to to be statistically significant (trough concentrations: *p* > 0.05, AUC: *p* > 0.05, Pearson test).

**Figure 4:**
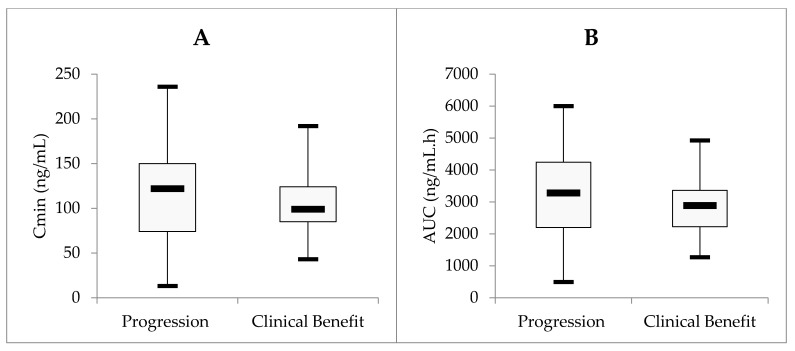
(**A**,**B**) Relationships between clinical issues and concentration trough levels (**A**) or drug exposure (**B**). No statistical difference was found in Cmin or AUC values between patients with clinical benefit and patients with progressive disease.

Regarding efficacy, mean trough concentrations at baseline were 107 ± 43 ng/mL and 111 ± 54 ng/mL in patients with clinical benefit and with progressive disease, respectively. Mean AUC_T, SS_ was 2721 ± 1029 ng/mL.h and 3021 ± 1353 ng/mL.h in patients with clinical benefit and with progressive disease, respectively. Neither Cmin nor AUC_T, SS_ was found to be associated with efficacy (*p* > 0.05, *t*-test; Figure 4).

### 2.6. Efficacy of Sunitinib Depending on Empirical Change in Dosing

**Figure 5:**
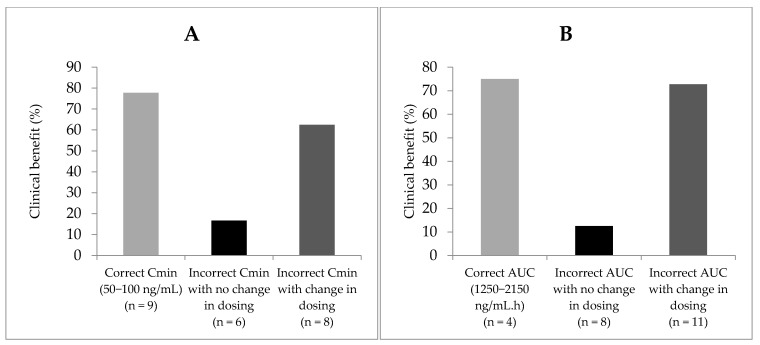
Clinical benefit in patients depending on their baseline exposure values. (**A**) Trough concentrations, (**B**) AUC and the subsequent change in dosing.

When considering patients with adequate Cmin exposure at baseline (i.e., between 50 and 100 ng/mL), 77.8% of them had clinical benefit upon RECIST evaluation at 3 months. When considering patients with baseline concentrations out of the trough concentrations with a subsequent empirical change in dosing, efficacy was 62.5%, whereas similar patients without a subsequent change in dosing showed only 16.7% clinical benefit. Similarly, patients with adequate AUC at baseline had 75% clinical benefit upon RECIST evaluation at 3 months, patients out of the target AUC range at baseline with a subsequent change in dosing had 72.7% clinical efficacy, whereas patients out of the AUC range and without dose adjustment had only 12.5% efficacy (Figure 5 and Table 3).

### 2.7. Model-Based Dosing Recommendations

Based upon target trough concentrations set at 50–100 ng/mL, the PK/PD model proposed to increase sunitinib dosing in three patients (9.7%), to decrease dosing in 19 patients (61.3%) and to keep initial dosing in nine patients (29%).

When AUC between 1200 and 2150 ng/mL.h was used as the target exposure, the model proposed to increase sunitinib dosing in two patients (6.5%), to decrease dosing in 26 patients (83.9%) and to keep initial dosing in three patients (9.7%).

Cmin or AUC targets led to similar recommendations in 74.2% of patients, but conflicting results in 25.8% of patients. As a consequence, the PK/PD model recommendations based upon AUC were always more protective for patients than recommendations based upon concentration trough level. Indeed, in seven patients, the model based upon target trough concentrations recommended to keep initial dosing whereas the model based upon AUC recommended a decrease in dosing. Similarly, increased dosing was suggested in one patient based upon trough concentrations, whereas the model based upon AUC proposed to keep initial dosing.

Furthermore, model-based recommendations would have led to decreased dosing in eight out of 11 patients (73%) who experienced grade 3 adverse events and 10 out of 11 (91%) based upon target trough concentrations and target AUC, respectively (see next paragraph).

Only two patients were considered as underexposed (one patient based upon trough concentrations below 50 ng/mL, one patient with both trough concentrations below 50 ng/mL and AUC below 1250 ng/mL.h). Model-based recommendations were to increase dosing from 50 mg to 87.5 and 100 mg QD, respectively.

### 2.8. Retrospective Comparison between Empirical and Model-Based Change in Dosing

**Figure 6:**
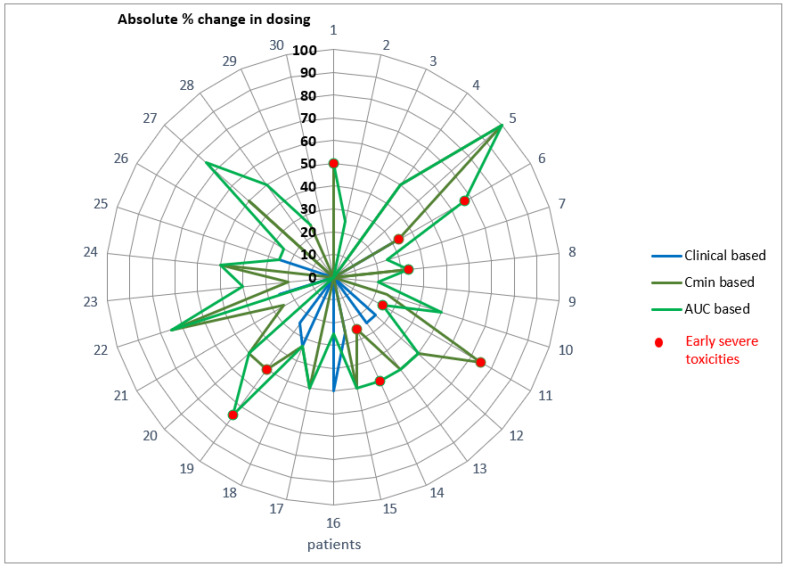
Comparison between model-based change in dosing (orange: based on Cmin, green: based on AUC) and clinical-based change in dosing (blue). Circle radius represents the absolute change in %, as compared with initial dosing (center at 0 = no change).

As shown in Figure 6, the model-based recommendations expressed as absolute % of the initial dosing suggested for both metrics (i.e., Cmin and AUC) that sunitinib dosing should be modified more frequently than with empirical decisions, especially for patients displaying early-onset grade 2 and grade 3 toxicities.

Details of baseline, empirical change or model-based changes in dosing are further given in Table 4 for the 17 patients with severe early-onset toxicities. Based upon clinical signs, sunitinib dosing was reduced in 7 out of 17 patients (41%) with severe toxicities whereas Cmin-based and AUC-based recommendations would have led to reduced dosing in 14 (82%) and 15 (88%) patients, respectively. Reduction in sunitinib dosing ranged from −20% to −50% as compared to the initial dosing when using Cmin, and from −20% to −75% when using AUC. Conversely, using Cmin as a metric would have led to wrongly increased dosing in two patients who showed severe toxicities, whereas using AUC as a metric would have led to wrongly increased dosing in one patient who had severe toxicities.

Regarding the only two patients with sunitinib levels below the target exposures at baseline, both were actually maintained at 50 mg based upon clinical examination. One patient had clinical benefit, whereas the other one eventually had progressive disease (Table 5).

Details of baseline, empirical change or model-based changes in dosing are further given in Table 6 for the 11 patients with progressive disease.

## 3. Discussion

Developing TDM with oral targeted therapies has been an appealing strategy to reduce inter-individual variability in PK due to multiple causes, such as DDI, genetic polymorphism (PGx), poor adherence or comorbidities.

PK/PD relationships with sunitinib have been extensively described. The dose-finding study of sunitinib was based upon target trough levels between 50 and 100 ng/mL for sunitinib and active metabolite N-desethyl sunitinib [3]. In a PK/PD meta-analysis, Houk et al. confirmed next that exposure (i.e., AUC) to sunitinib + N-desethyl sunitinib was associated with clinical outcome in patients with gastro-intestinal tumors (GIST) or mRCC [12].

Bertolaso et al. have demonstrated that even if TDM is not routinely used for sunitinib in every mRCC patient, pharmacokinetically guided dosing could be useful for frail individuals such as patients with cardiac transplant [13]. Several PK/PD models have been further proposed as a means to predict the efficacy of sunitinib. For instance, Diekstra et al. have proposed a comprehensive PK/PD/PGx model for sunitinib in both mRCC and metastatic colorectal cancer (mCRC) patients. They found that drug exposure was related to efficacy in mCRC patients but, surprisingly, in mRCC patients, monitoring basal levels of sVEGFR2 was more useful than PK to forecast sunitinib efficacy [14]. Similarly in GIST patients treated with sunitinib, modeling early changes in standardized uptake value upon PET scan imaging helped to predict survival [15]. In another study, Narjoz et al. found that lean body weight and genetic polymorphisms on the ABCG2 transporter contributed to the PK variability of sunitinib, and that AUC above 1950 ng/mL.h was associated with prolonged survival in mRCC patients [16]. Here, inter-patient variability in exposure levels ranged from 42% to 45% for AUC and Cmin, respectively. Ideally, TDM should help in customizing dosing in a time-effective manner when patients are not in the right therapeutic window. Indeed, TDM can provide early and relevant information on inadequate exposure levels before clinical signs (e.g., side effects or lack of efficacy) actually show in patients. Implementing TDM with adaptive dosing in routine practice in oncology remains difficult and most oncologists prefer to rely on their clinical judgment, rather than using model-based dosing recommendations [17]. Logistic considerations such as strict respect for sampling time (i.e., T24 h) to measure trough concentrations have long been hard requirements to meet in routine clinical practice. Vagueness of subsequent dosing recommendations or the difficulties in establishing appropriate target concentrations could also be limitations. However, the rise in oral targeted therapies has shown that drug exposure is correlated with clinical outcome in several settings [18,19] In this respect, PK/PD modeling offers valuable help. First, the Bayesian estimate of individual PK parameters from sparse samples can help in simulating trough concentrations, regardless of the exact time of the sampling. Indeed, once individual PK parameters have been calculated with a good-quality estimates, it is possible to determine in silico the trough concentrations and the resulting AUC. Second, provided that the therapeutic window has been identified, the model can then calculate the exact dosing to achieve appropriate exposure [20]. Here, we have tested such a PK/PD model implemented in Monolix^®^ to monitor sunitinib and N-desethyl sunitinib levels in mRCC patients and retrospectively propose dosage adjustment if required, based upon two distinct metrics: trough concentrations and plasma AUC. The data we have collected here show how using a PK model helps in simulating trough concentrations when drawing blood samples is not feasible at 24 h. As in routine oncology, sampling the patients precisely at the required time can be difficult, especially with ambulatory patients treated with oral targeted therapies, so using a PK model is a valuable strategy, giving much flexibility to perform TDM. For instance, here only 19.4% of the samples were withdrawn precisely at T24 h, but the model was still able to calculate individual PK parameters and simulate virtual trough concentrations. Overall, 54.8% of patients were found to be inadequately exposed with respect to the target trough concentrations of sunitinib and 74.2% of patients were inadequately exposed with respect to the target AUC. Although not statistically significant because of the small numbers of patients, a trend was observed between both Cmin and AUC and early-onset toxicities, i.e., the higher the exposure, the greater the side effects. This observation calls for the implementation of TDM with sunitinib since most of the patients treated with standard dosing failed to be within the target exposure levels. Our observation is in line with clinical observations by Noda et al., who showed that severe toxicities in mRCC patients were associated with elevated residual concentrations of sunitinib and N-desethyl sunitinib [21]. Regarding patients who were not in the therapeutic window, model-based recommendations for tailored dosing ranged from 12.5 to 100 mg, i.e., a −75% to + 100% change as compared with initial standard dosing. The discrepancy between Cmin-based or AUC-based recommendations, as explained in the “model simulation” section below, were mainly due to the fact that simulated AUCs were always slightly higher than simulated trough concentration values. Of note, at the bedside, a change in dosing was primarily left to the clinician’s choice, i.e., based upon clinical observations, and not upon exposure levels or subsequent model’s proposals for customized dosing. Interestingly, and although numbers were small, patients with correct baseline exposure had an 80% response rate eventually—whereas patients who were not correctly exposed at baseline had response rate of only about 22.2%, i.e., 3.5 times lower. Very interestingly, the subset of patients with inadequate sunitinib exposure at baseline for whom doses were changed in an empirical manner showed a 72.7% response rate, i.e., close to the values of the patients with the right exposure levels upon standard dosing. This suggests that exposure could be a critical factor for sunitinib efficacy, and that increasing dosing could be an actionable item to improve response rates in patients with low drug levels. Of note, the fact that in this study, globally, no relationship between baseline exposure (i.e., Cmin or AUC) and clinical efficacy was found can be partly explained by the fact that, here, drug exposure was solely measured at baseline, and not after subsequent empirical changes in dosing. As efficacy was evaluated 3 months after that treatment had started, the subsequent changes in dosing in 53.8% of patients were thus a major confounding factor when trying to find a relationship between initial drug exposure and clinical outcome 3 months later. In this respect, here, the collected data on efficacy and the possible usefulness of TDM plus adaptive dosing to improve efficacy cannot be conclusive, an observation already reported by others for mRCC patients [14]. This calls for implementing longitudinal monitoring of sunitinib concentrations, especially when patients are likely to have their dosing changed over time. In this real-world study with sunitinib, we observed that actual changes in dosing were not as drastic and as frequent as compared with what the PK/PD model would have recommended at baseline. This observation is fully in line with clinical reports on routine TDM with other oral targeted therapies published by others [13,17,18,19,20,21]. Despite several flaws related to its single-institute nature and the limited number of patients, plus the fact that no predictive performance validation step could be run, this study presents several findings. First, although not significant, a trend between exposure levels and severe early-onset toxicities was observed. Importantly, the vast majority (82–88%) of the patients who experienced those severe toxicities would have had their dosing reduced following model recommendations, based upon measurement of baseline exposure levels of sunitinib and N-desethyl sunitinib. This suggests that the incidence of treatment-related toxicities could be reduced by implementing TDM-based, model-driven adaptive dosing with sunitinib.

## 4. Materials and Methods

### 4.1. Patients

**Figure 7:**
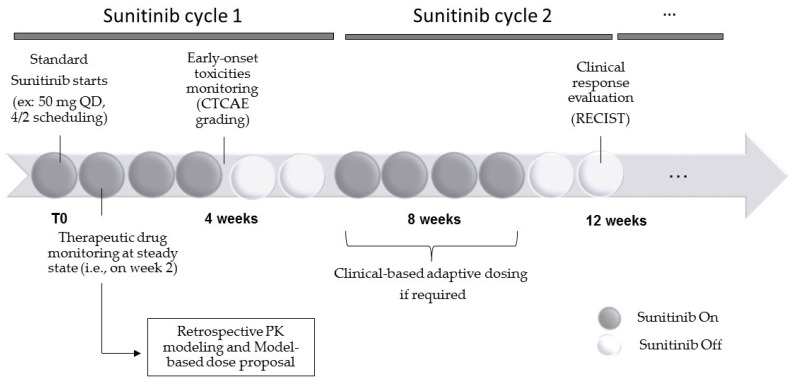
Generic time line of the study.

All routine patients with a diagnosis of metastatic RCC of any histology and scheduled for sunitinib treatment from 2017 to 2020 in our institute were considered for monitoring. All patients were treated with standard sunitinib in the Medical Oncology Unit at La Timone University Hospital of Marseille. Patients were identified from the hospitals’ administrative database, and relevant information regarding empirical changes in dosing, efficacy and toxicity endpoints were retrieved from electronic medical records. All patients underwent therapeutic drug monitoring (TDM) as part of routine care in our institute with oral targeted therapies. Data exploitation after anonymization was granted upon the non-opposition principle at the Assistance Publique Hôpitaux de Marseille institute (i.e., unless the contrary is stated, all data collected during routine care can be used for biomedical research). A time line of the study is provided in Figure 7.

### 4.2. Measurement of Plasma Concentration of Sunitinib and N-Desethyl Sunitinib

Blood samples for monitoring sunitinib concentrations were taken at steady state (i.e., at least 10 days after the beginning of the treatment or after any change in dosing), ideally immediately before the next administration (i.e., trough concentrations). When sampling time did not meet this criterion, PK modeling helped to simulate virtual trough concentrations (see PK/PD modeling below). Sunitinib and N-desethyl sunitinib were both assayed after s simple precipitation step using a liquid chromatography tandem mass spectrometry technique validated following ISO-1589 and EMEA guidelines [22]. LOQ was 10 ng/mL for both compounds. The sum of sunitinib and N-desethyl sunitinib was defined as total sunitinib concentration, either to determine trough concentrations or AUC (see PK/PD modeling below).

### 4.3. Sunitinib Dosing and Adaptive Dosing

All patients were treated following standard dosing (i.e., 50 mg QD on a 4/2 or 2/1 scheduling basis) at treatment initiation, except 2 patients who were treated at 62.5 mg QD on a 2/1 basis, 3 at 37.5 mg QD on a 4/2 basis and another one who was treated at 12.5 mg QD on a 4/2 basis. Next, change in dosing was left to the oncologist’s consideration, based upon clinical signs (e.g., toxicities or lack of efficacy) or results from TDM. In parallel, PK/PD modeling was performed based upon observed TDM values and alternate dosing based upon target AUC or target trough concentrations were simulated.

### 4.4. Sampling for Therapeutic Drug Monitoring

All patients were sampled at steady state, i.e., usually 10 days after treatment was initiated. Sunitinib concentrations were only analyzed at baseline, and no longitidunal monitoring of sunitinib concentrations was possible, even after dosing was subsequently changed. Only 6 patients (19.4%) were precisely sampled on their actual trough concentrations (i.e., T24H), with the rest of the group being sampled earlier (e.g., over a 2–12 h period) or later (e.g., at T25-26H). When sampling times did not precisely match the trough concentrations, a simulated T24h concentration was then calculated in silico by the PK model implemented on Monolix^®^ (see the methods section below).

### 4.5. PK/PD Modeling

The pharmacokinetics of sunitinib was already extensively described in the literature by Houk et al., for both the parent drug and its primary active metabolite [12]. In Houk et al., separate models were developed for sunitinib and active SU12662 metabolite, but both shared the same structural framework, i.e., a two-compartment model with first-order absorption and elimination. Values of final parameter estimates for both the base model and the model with covariates for sunitinib and SU12662 were made available, but we finally chose to use the base model for two practical reasons. First, some of the covariates in the Houk et al. model were not necessarily available in real-world settings. Secondly, we made a comparison between the dosing recommendations with and without the selected covariates, and there was no significant difference in the final dosing (data not shown). Here, population modeling allowed for describing the time course of sunitinib and its active metabolite in plasma, considering the inter-individual variability. The population model provides the statistical distribution of each PK parameter. By further implementing the measurement information (both parent drug and metabolite concentrations in plasma), we could compute then the conditional distribution corresponding to the statistical law with respect to the measured concentrations [23]. We thus did not compute the empirical Bayesian estimate (EBE) which corresponds to the most probable values, but we rather computed PK parameters that were representative of each individual. We then did not focus on the most probable value, but rather on all the values that made sense for the individual. This represented the uncertainty of the individual PK parameter values. Thus, instead of having the PK parameters of any patient in the population, we could estimate the pharmacokinetic parameters of the patient for whom plasma concentration was known within the population. This allowed for having a much lower uncertainty when determining his/her parameters, thus eventually improving the quality of the estimates.

Using these PK parameters enabled simulating several dosing regimens (i.e., from 25 mg to 100 mg by 12.5 mg steps) and seeing the percentage of PK profiles which were in the required therapeutic range defined in [3,12]. To do that, we used the PK parameters from the conditional distribution to compute the concentrations of both sunitinib and its active metabolite. Then, we could compute the associated trough values at steady state and finally we were able to estimate for each vector of parameters the steady state trough concentration and the corresponding AUC. Here, the sunitinib model was calibrated to reach two distinct target exposures: trough concentrations between 50 and 100 ng/mL [3], or AUC between 1200 and 2150 ng/mL.h [12].

Based on this information, we proposed a metric of the percentage of how many simulated profiles were in the window for each dose regimen. This information could thus be used to customize the dosing for a specific patient, provided that at least one plasma concentration for sunitinib and its active metabolite were made available, regardless of the sampling time.

This model for adaptive dosing was implemented in Monolix^®^ (Lixoft, France) and made available online using a Shiny application.

### 4.6. Clinical Endpoints

Treatment modification included the numbers and proportions of patients who had dose modification (i.e., increase or decrease), discontinued treatment or those who switched to another line of treatment regardless of the cause. Response was evaluated following standard RECIST criteria in mRCC. The clinical benefit was a regression of the tumor size (i.e., partial or complete response) or non-progression of the tumor (i.e., stable disease) over the observation period.

Toxicity was graded according to standard CTCAE 6.0. criteria. Safety outcomes included the numbers and proportions of patients who presented adverse events (AEs). With respect to the daily dosing of sunitinib, severe toxicities were defined as grade 2 or greater side effects as collected from electronic medical records.

### 4.7. Statistical Analysis

All statistical analyses were performed using MedCalc software 4.1. (Belgium). Depending on data distribution (i.e., normal distribution, Kolmogorov–Smirnov testing) and equal variance testing, a *t*-test or non-parametric Pearson test were considered for statistical analysis. Typically, Pearson testing was performed when normality or equal variance testing failed, or when statistical power below 0.8 prevented a *t*-test from being run. Pre-checking for normality, equal variance or statistical power was performed with Sigma Stat 2.1.(SPPS, Germany). A *p* value of 0.05 was regarded as statistically significant.

## 5. Conclusions

Precision medicine in oncology is a generic term encompassing several strategies to tailor treatment in patients with cancer—mostly as an effort to select the drugs which are the most likely to be efficacious. Here, we have studied in a real-world setting to what extent TDM plus model-based adaptive dosing could help in customizing sunitinib dosing. Whereas the impact on efficacy remains to be fully investigated, data on toxicity suggest that model-based dosing could have helped in reducing the incidence of side effects. Overall, this real-world study suggests that exposure matters and that dosing could be an actionable item to improve clinical outcome. In this respect, TDM with subsequent modeling could be a valuable tool for decision making in patients with mRCC treated with sunitinib.

## Figures and Tables

**Table 1:** Patients’ baseline demographics and clinical characteristics.

	No. (%)
Characteristic	Patients (n = 31)
Age, years	
Median	62
Range	26–87
Sex	
Male	26 (83.9)
Female	5 (16.1)
Body weight (kg)	
Median	78
Range	46–160
BSA (m^2^)	
Mean	1.9
Standard deviation	±0.2
BMI (kg/m^2^)	
Mean	25.6
Standard deviation	±7.2
Histology	
Clear cell	31 (100)
Metastasis	27 (93.1)
Initial dosing	
62.5 mg QD	2 (6.4)
50 mg QD	25 (80.7)
37.5 mg QD	3 (9.7)
25 mg QD	0 (0)
12.5 mg QD	1 (3.2)
Schedule	
4 weeks ON/2 weeks OFF	29 (93.6)
2 weeks ON/2 weeks OFF	1 (3.2)
Other	1 (3.2)

Abbreviations: BSA, Body Surface Area; BMI, Body Mass Index; QD, Quaque Die.

**Table 2:** Adverse events graded according to standard CTCAE 6.0 criteria.

	Patients (n = 27)		
	n (%)		
Adverse Event	No Toxicity	Grade 1/2	Grade 3
Diarrhea	2 (7.4)	20 (74.1)	5 (18.5)
Arterial hypertension	8 (29.6)	15 (55.6)	4 (14.8)
Skin toxicity	11 (40.7)	14 (51.9)	2 (7.4)
Headache, anosmia	18 (66.7)	9 (33.3)	0 (0)
Fatigue	8 (29.6)	17 (63)	2 (7.4)
Neutropenia	24 (89.9)	2 (7.4)	1 (3.7)

**Table 3:** Clinical benefit as measured by RECIST criteria at 3 months in patients depending on their baseline exposure values (**A**: target = trough concentrations, **B**: target = AUC) with or without change in dosing.

**A**	**Adequate Trough concentrations (50–100 ng/mL)**	**Abnormal trough concentrations with subsequent change in dosing**	**Abnormal trough concentrations without subsequent change in dosing**
**n**	9	8	6
**Clinical benefit n (%)**	7 (77.8)	5 (62.5)	1 (16.7)
**B**	**Adequate AUC (1250–2150 ng/mL.h)**	**Abnormal AUC with subsequent change in dosing**	**Abnormal AUC without subsequent change in dosing**
**n**	4	11	8
**Clinical benefit n (%)**	3 (75)	8 (72.7)	1 (12.5)

**Table 4:** Comparison between clinical-based change in dosing and proposed change in dosing by the model based upon Cmin or AUC considerations. 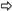: no change, 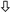: decrease, 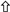: increase.

Early-Onset Toxicities (n = 17 Patients)			
Initial Dosing (mg)	Clinical-Based Dosing (% Change)	Cmin-Based Dosing (% Change)	AUC-Based Dosing (% Change)
50	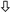 37.5 (−25%)	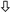 25 (−50%)	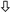 25 (−50%)
50	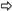 50 (0%)	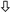 25 (−50%)	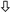 25 (−50)
37.5	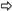 37.5 (0%)	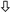 25 (−33%)	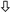 25 (−33%)
62.5	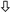 50 (−20%)	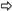 62.5 (0%)	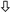 50 (−20%)
50	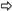 50 (0%)	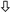 37.5 (−25%)	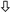 25 (−50%)
50	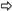 50 (0%)	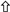 87.5 (+75%)	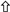 62.5 (+25%)
50	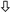 37.5 (−25%)	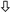 25 (−50%)	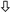 25 (−50%)
50	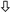 37.5 (−25%)	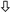 25 (−50%)	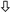 25 (−50%)
50	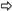 50 (0%)	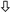 37.5 (−25%)	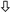 25 (−50%)
50	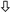 37.5 (−25%)	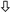 25 (−50%)	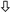 25 (−50%)
50	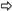 50 (0%)	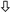 25 (−50%)	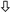 25 (−50%)
50	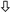 37.5 (−25%)	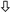 25 (−50%)	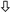 12.5 (−75%)
50	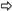 50 (0%)	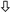 25 (−50%)	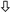 25 (−50%)
50	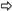 50 (0%)	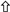 62.5 (+25%)	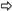 50 (0%)
50	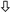 37.5 (−25%)	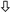 12.5 (−75%)	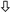 12.5 (−75%)
62.5	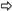 62.5 (0%)	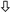 50 (−20%)	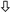 37.5 (−40%)
50	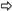 50 (0%)	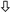 25 (−50%)	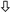 25 (−50%)

**Table 5:** Comparison between clinical-based change in dosing and proposed change in dosing by the model in the two patients with baseline exposures below the targets.

Initial Dosing	Trough Levels (Target: 50–100 ng/mL)	AUC (Target: 1250–2150 ng/mL.h)	Model-Based Recommendation	Clinical-Based Recommendation	Clinical Outcome	Early-Onset Toxicities
50 mg	13	491	100 mg	50 mg	Progressive Disease	No
50 mg	43	1264	87.5 mg	50 mg	Clinical Benefit	No

**Table 6:** Comparison between clinical-based change in dosing and proposed change in dosing by the model based upon Cmin or AUC considerations for patients with progressive disease. 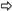: no change, 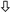: decrease, 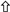: increase.

Patients with Progressive Disease (n = 11)				
Initial Dosing (mg)	Clinical-Based Dosing (mg)	Cmin-Based Dosing (mg)	AUC-Based Dosing (mg)	Early Severe Toxicities
50	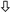 37.5 (−25%)	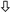 25 (−50%)	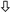 25 (−50%)	yes
50	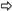 50 (0%)	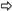 50 (0%)	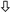 37.5 (−25%)	no
50	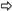 50 (0%)	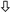 25 (−50%)	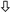 25 (−50%)	yes
50	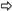 50 (0%)	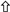 100 (+100%)	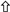 100 (+100%)	no
62.5	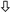 50 (−20%)	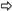 62.5 (0%)	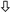 50 (−20%)	yes
50	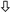 37.5 (−25%)	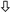 25 (−50%)	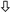 25 (−50%)	yes
50	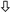 25 (−50%)	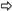 50 (0%)	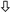 37.5 (−25%)	no
50	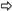 50 (0%)	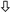 25 (−50%)	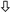 25 (−50%)	no
50	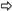 50 (0%)	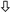 25 (−50%)	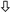 25 (−50%)	yes
50	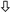 37.5 (−25%)	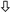 12.5 (−75%)	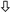 12.5 (−75%)	yes
62.5	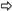 62.5 (0%)	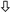 50 (−20%)	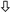 37.5 (−40%)	yes

## Data Availability

All raw clinical and PK data are made available upon request at ciccolini.joseph@gmail.com.
